# Methicillin-Susceptible, Vancomycin-Resistant *Staphylococcus aureus*, Brazil

**DOI:** 10.3201/eid2110.141914

**Published:** 2015-10

**Authors:** Diana Panesso, Paul J. Planet, Lorena Diaz, Jean-Emmanuel Hugonnet, Truc T. Tran, Apurva Narechania, Jose M. Munita, Sandra Rincon, Lina P. Carvajal, Jinnethe Reyes, Alejandra Londoño, Hannah Smith, Robert Sebra, Gintaras Deikus, George M. Weinstock, Barbara E. Murray, Flavia Rossi, Michel Arthur, Cesar A. Arias

**Affiliations:** Universidad El Bosque, Bogota, Colombia (D. Panesso, P.J. Planet, L. Diaz, J.M. Munita, S. Rincon, L.P. Carvajal, J. Reyes, G. M. Weinstock, C.A. Arias);; University of Texas Medical School, Houston, Texas, USA (D. Panesso, T.T. Tran, J.M. Munita, A. Londoño, B.E. Murray, C.A. Arias);; American Museum of Natural History, New York (P.J. Planet, A. Narechania);; Columbia University, New York, New York, USA (P.J. Planet, A. Londoño, H. Smith);; Centre de Recherche des Cordeliers, Paris, France (J.E. Hugonnet, M. Arthur);; Université Pierre et Marie Curie, Paris (J.E. Hugonnet, M. Arthur);; Université Paris Descartes, Paris (J.E. Hugonnet, M. Arthur);; Clinica Alemana de Santiago, Santiago, Chile (J.M. Munita);; Universidad del Desarrollo, Santiago (J.M. Munita);; Icahn School of Medicine at Mount Sinai, New York (R. Sebra, G. Deikus);; The Jackson Laboratory for Genomic Medicine, Farmington, Connecticut, USA (G.M. Weinstock);; Universidade da São Paulo, São Paulo, Brazil (F. Rossi)

**Keywords:** Staphylococcus aureus, bacteria, antimicrobial resistance, methicillin susceptibility, vancomycin resistance, methicillin-susceptible, vancomycin-resistant, methicillin-susceptible S. aureus, MSSA, bacterial infections, bacteremia, gram-positive bacterial infections, Brazil

## Abstract

We report characterization of a methicillin-susceptible, vancomycin-resistant bloodstream isolate of *Staphylococcus aureus* recovered from a patient in Brazil. Emergence of vancomycin resistance in methicillin-susceptible *S. aureus* would indicate that this resistance trait might be poised to disseminate more rapidly among *S. aureus* and represents a major public health threat.

Acquisition of high-level vancomycin resistance by *Staphylococcus aureus* represents a major public health risk because this antimicrobial drug continues to be the first-line and most inexpensive therapy to treat methicillin-resistant *S. aureus* (MRSA) despite concerns about its clinical efficacy. Recently, we described vancomycin-resistant MRSA (VR-MRSA) recovered from the bloodstream of a patient in Brazil (*1*). VR-MRSA belongs to sequence type (ST) 8 and is phylogenetically related to the community-associated (CA) MRSA USA300 genetic lineage that has rapidly disseminated in the United States and the northern region of South America (USA300-Latin American variant [USA300-LV]) (*1**,**2*). The *vanA* gene cluster in VR-MRSA was carried by a transferable staphylococcal plasmid (pBRZ01). We characterized a clinical isolate of vancomycin-resistant, methicillin-susceptible *S. aureus* (VR-MSSA) and document the in vivo transfer of the *vanA* gene cluster to 2 unrelated *S. aureus* strains causing bacteremia within the same patient.

## The Study

On August 28, 2012, a blood culture from a patient in Brazil was reported positive for 2 isolates of MSSA while the patient was receiving daptomycin therapy (Technical Appendix). One MSSA isolate was susceptible to all antimicrobial drugs tested (VS-MSSA). The second isolate (VR-MSSA) had a vancomycin MIC of 256 µg/mL and was also resistant to gentamicin (Table 1). Both isolates were susceptible to daptomycin (MIC 0.5 μg/mL). Thirteen days earlier, 2 MRSA isolates, 1 of which was resistant to vancomycin (VR-MRSA), were recovered from the blood of the same patient (Technical Appendix) (*1*). The daptomycin MICs for both MRSA strains were also 0.5 μg/mL.

**Table 1:** *Staphylococcus aureus* strains used in analysis of methicillin and vancomycin resistance, Brazil*

Strain	Strain characteristics	MIC, μg/mL		Reference
		Vancomycin	Gentamicin	
VS-MRSA	Isolated from the bloodstream of a patient in Brazil	0.5	0.5	(*1*)
VR-MRSA	Isolated from the blood of the same patient above and carrying *vanA*-containing pBRZ01	>256	32	(*1*)
VS-MSSA	Isolated from the blood of the same patient 13 d after isolation of VR-MRSA	1	0.75	This study
VR-MSSA	Isolated from the same blood culture as VS-MSSA	256	48	This study
RN4220-RF	Laboratory strain of *S.aureus* used as recipient for mating experiments; fusidic acid and rifampin-resistant	1	1	(*1*)
Transconjugant 1†	Transconjugant obtained from a mating experiment using VR-MSSA as donor and VS-MRSA as recipient	>256	48	This study
Transconjugant 2†	Transconjugant obtained from a mating experiment using VR-MRSA as donor and VS-MSSA as recipient	>256	64	This study
Transconjugant 3†	Transconjugant obtained from a mating experiment using VR-MSSA as donor and RN4220-RF as recipient	>256	64	This study

*VS-MRSA, vancomycin-susceptible, methicillin-resistant *S. aureus*; VS-MSSA, vancomycin-susceptible, methicillin-susceptible *S. aureus*; VR-MSSA, vancomycin-resistant, methicillin-susceptible *S. aureus*. †A fusidic acid–resistant derivative was generated for mating experiments. All mating experiments were performed on brain heart infusion (BHI) agar in the presence of vancomycin (32 µg/mL) and fusidic acid (25 µg/mL) to select for transconjugants.

Bacterial strains used in this study (Table 1) were grown in brain–heart infusion broth and agar. Plasmid pBRZ01 was transferred by using filter mating (*3*) and VR-MSSA and VR-MRSA as donors and VS-MSSA, VS-MRSA, and RN4220RF as recipients (Table 1). Transconjugants were selected on brain heart infusion medium containing vancomycin (32 µg/mL) and fusidic acid (25 µg/mL). Colonies from each mating experiment were subjected to digestion with *Sma*I and pulsed-field gel electrophoresis to investigate genetic relatedness (*1*). Plasmids carrying the *vanA* gene cluster were detected by using S1 nuclease digestion followed by hybridization with a *vanA* probe (*4*).

Whole-genome sequencing of VR-MSSA, VS-MSSA, and 2 representatives of the Chilean/Cordobes clone (M1, M91) was performed by using MiSeq PacBio RS II (Illumina, San Diego, CA, USA) to close the VR-MSSA genome (*5*) (online Technical Appendix). Phylogenetic analysis was performed by using the maximum-likelihood framework within RAxML v7.4.2 (*6*). For cell wall analysis, extraction and separation of peptidoglycan precursors was performed as described (*7*).

**Figure 1:**
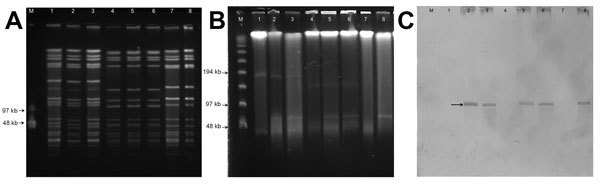
Molecular typing of *Staphylococcus aureus* strains, Brazil. A) *Sma*I digestion of total DNA, followed by pulsed-field gel electrophoresis. Lane M, lambda ladder (molecular masses are indicated in kilobases on the left); lane 1, vancomycin-susceptible, methicillin-resistant *S. aureus* (VS-MRSA) isolated from the blood of a Brazilian patient (*1*); lane 2, vancomycin-resistant MRSA (VR-MRSA) isolated from the same patient and blood culture (*1*); lane 3, transconjugant 1 obtained from a mating experiment using vancomycin-resistant MSSA (VR-MSSA) as donor and VS-MRSA as recipient; lane 4, vancomycin-susceptible MSSA (VS-MSSA) isolated from the blood of the same patient 13 days after isolation of VR-MRSA; lane 5, VR-MSSA isolated at the same time as VS-MSSA; lane 6, transconjugant 2 obtained from a mating experiment using VR-MRSA as donor and VS-MSSA as recipient; lane 7, *S. aureus* RN4220 RF, lane 8, transconjugant 3 obtained using VR-MSSA as donor and RN4220 RF as recipient. B) S1 digestion of total DNA using the same strains shown in panel A. C) Hybridization with *vanA* probe using the same strains shown in panel A. Arrow indicates a positive signal for the *vanA* gene.

The PFGE patterns of both isolates (VR-MSSA and VS-MSSA) were indistinguishable, and in vitro growth rates were similar (Figure 1, panel A). S1 nuclease analyses indicated that VR-MSSA harbored a plasmid of ≈55 kb, which yielded a positive result when hybridized with a *vanA* probe (Figure 1, panels B, C) and was similar in size to the previously described *vanA*-containing plasmid pBRZ01 identified in the same patient (*1*). pBRZ01 of VR-MSSA was readily transferred to *S. aureus* RN4220-RF (efficiency = 3 × 10^−5^/donor). In vitro conjugative transfer of pBRZ01 between MRSA and MSSA strains recovered from the patient’s bloodstream was also readily achieved with efficiencies ranging from 4.3 × 10^−7^/donor to 2.5 × 10^−6^/donor. Acquisition of the pBRZ01 by corresponding strains resulted in resistance to vancomycin and gentamicin (Table 1).

Genome sequencing (Technical Appendix) showed that VR-MSSA and VS-MSSA belong to clonal complex (CC) 5 (sequence type ST5) and harbor staphylococcal protein A (Spa) type t002. VS-MSSA and VR-MSSA have the characteristic CC5 genetic traits described by Kos et al. (*8*). The genome of VR-MSSA has a 2,906,602-bp chromosome and 3 extrachromosomal elements, including a plasmid of 55,713 bp identical to the previously described *vanA*-carrying pBRZ01 (*1*), which also harbors *aac(6′)-aph(2′′)*, which confers gentamicin resistance.

**Figure 2:**
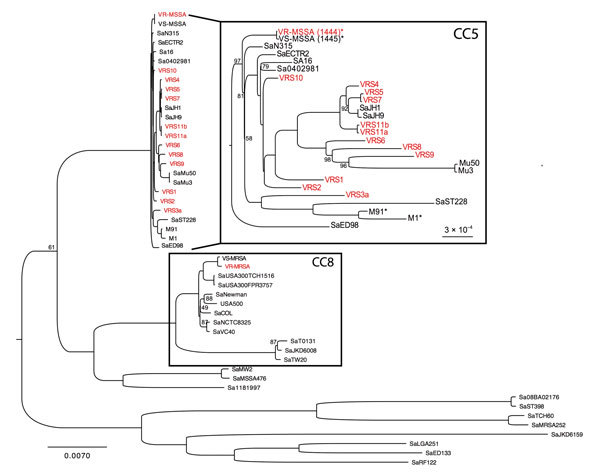
Phylogenetic analyses of *Staphylococcus aureus* strains, Brazil. Whole-genome phylogenetic tree (dataset = 325,732 single-nucleotide polymorphisms, gamma-based log likelihood − 1909607.06950) of the *S. aureus* species showing position of vancomycin-resistant, methicillin-susceptible *S. aureus* (VR-MSSA) and vancomycin-susceptible MSSA (VS-MSSA) isolates sequenced for this study. Vancomycin-resistant S. *aureus* (VRSA) strains are shown in red. Numbers on branches are bootstrap values based on 1,000 resampling iterations. All branches without numbers had bootstrap values of 100%. Branch lengths are proportional to number of nucleotide substitutions per site (scale bars). Inset labeled CC5 is expanded to emphasize the polyphyly of VRSA strains. *Genomes sequenced for this study. M1 and M91 are members of the Chilean/Cordobes clone that is widespread in Latin America (Technical Appendix). CC, clonal complex.

Comparison of the core genomes of VR-MSSA and VS-MSSA showed only 20 single-nucleotide polymorphism differences, which suggested a close genetic relationship and probably representing the same organism that acquired pBRZ01. Phylogenetic analysis (Figure 2) confirmed that VR-MSSA is not a derivative of VR-MRSA (*1*) (isolated days before from the same patient) and emphasized the relationship of this strain to other vancomycin-resistant *S. aureus* and MRSA isolates with intermediate susceptibility to vancomycin (VISA).

We analyzed the pool of cytoplasmic peptidoglycan precursors of VR-MSSA grown in the absence or presence of 50 μg/mL of vancomycin for induction of the *vanA* cluster (Table 2). Tandem mass spectrometry analysis identified 3 nucleotide precursors ending in d-alanyl-d-alanine (UDP-MurNAc-pentapeptide), d-alanyl-d-lactate (UDP-MurNAc-pentadepsipeptide), and d-Ala (UDP-MurNAc-tetrapeptide). Upon induction with vancomycin, UDP-MurNAc-pentapeptide was not detected, and UDP-MurNAc-pentadepsipeptide accounted for most of the precursors (Table 2). These results indicate that the *van*-encoded enzymes required for incorporation of d-Lac into the precursors were fully functional in VR-MSSA. Our results also show that the *vanA* cluster was inducible by vancomycin in the *S*. *aureus* host because only a small proportion of the precursors (4%) ended in d-Lac in the absence of the drug.

**Table 2:** Relative abundance of peptidoglycan precursors in *Staphylococcus aureus* strains, Brazil*

Precursor	Monoisotopic mass		Abundance (%) in corresponding strains		
	Observed	Calculated	VS-MSSA	VR-MSSA (not induced)	VR-MSSA (induced)
UDP-MurNaAc-tetrapeptide	1,078.35	1,078.35	27	32	29
UDP-MurNAc-pentapeptide	1,149.37	1,149.39	73	64	ND
UDP-MurNAc-pentadepsipeptide	1,150.37	1,150.37	ND	4	71

*Bacteria were grown on brain heart infusion (BHI) broth (not induced) or BHI supplemented with vancomycin (50 μg/mL). VS-MSSA, vancomycin-susceptible, methicillin-susceptible *S. aureus*; VR-MSSA, vancomycin-resistant, methicillin-susceptible *S. aureus;* ND, not detected.

Analyses of cell wall muropeptides from VR-MSSA showed 2 modifications of the l-Ala^1^-γ-d-Glu^2^-l-Lys^3^- d-Ala^4^-d-Ala^5^ stem peptide that are highly conserved in *S. aureus* strains, namely the amidation of the α-carboxyl of d-Glu^2^ to form d-iGln^2^ and the addition of a pentaglycine side chain on the ε-amino group of l-Lys^3^ by the Fem amino-acyltransferases (*9*). Induction of the *vanA* gene cluster led to 2 major modifications. First, stem peptides ended in d-Ala^4^, indicating that the peptidyl- d-Ala^4^- d-Ala^5^ target of vancomycin, and d-Ala^4^-d-Lac^5^ termini, were fully eliminated. Second, the pentaglycine side chain was frequently missing (Technical Appendix), indicating that replacement of d-Ala by d-Lac at the extremity of peptidoglycan precursors might have impaired the ability of Fem transferases to add Gly on l-Lys^3^.

## Conclusions

In this study, we demonstrated that the *vanA*-containing pBRZ01 plasmid previously described in MRSA was acquired by an invasive MSSA isolate within the same patient. Our findings also suggest that a *vanA*-containing plasmid (pBRZ01) was horizontally acquired at least twice during a short period by distinct *S. aureus* lineages within the same host (MRSA belonging to ST8 and an ST5 MSSA). VR-MSSA belongs to the ST5 lineage of CC5, a major hospital-associated lineage (*10*). The prevalent hospital-associated lineages circulating in Brazil are ST5 (New York/Japan and Pediatric clones), ST239 (Brazilian clone) and ST1 (USA400 clone) (*11*), and recent epidemiologic data showed replacement of the endemic Brazilian (ST239) clone by ST5 strains (*11**–**13*). Moreover, VR-MSSA is related to ST5 vancomycin-resistant *S. aureus* strains recovered in the United States (*8*) and to VISA isolates, including Mu50 and the hetero-VISA strain Mu3, initially recovered in Japan (*14*). It remains unclear why CC5 strains appear more likely to exhibit vancomycin resistance.

Our biochemical analysis indicates that the *vanA* gene cluster is fully functional in VR-MSSA, which leads to vancomycin-inducible production of d-Lac ending precursors and elimination of d-Ala- d-Ala containing peptidoglycan, as found in the enterococci (*15*). Our results also revealed a defect in side chain synthesis, although this did not prevent the synthesis of a functional and highly cross-linked peptidoglycan in VR-MSSA.

In summary, we report the in vivo acquisition of high-level vancomycin resistance in a bloodstream MSSA isolate. Of note, *vanA*-containing pBRZ01 was maintained even after the selective pressure of vancomycin had been removed, raising serious concerns about the possibility of further spread of resistance to this agent. However, no other MSSA strains containing this plasmid have been isolated so far in Brazil.

Technical Appendix Analysis of methicillin-susceptible, vancomycin-resistant *Staphylococcus aureus*, Brazil.

## References

[1] Rossi F, Diaz L, Wollam A, Panesso D, Zhou Y, Rincon S, Transferable vancomycin resistance in a community-associated MRSA lineage. N Engl J Med. 2014;370:1524–31. 10.1056/NEJMoa1303359 24738669 PMC4112484

[2] Reyes J, Rincon S, Diaz L, Panesso D, Contreras GA, Zurita J, Dissemination of methicillin-resistant *Staphylococcus aureus* USA300 sequence type 8 lineage in Latin America. Clin Infect Dis. 2009;49:1861–7. 10.1086/648426 19911971 PMC2787674

[3] Tomita H, Pierson C, Lim SK, Clewell DB, Ike Y. Possible connection between a widely disseminated conjugative gentamicin resistance (pMG1-like) plasmid and the emergence of vancomycin resistance in *Enterococcus faecium.* J Clin Microbiol. 2002;40:3326–33. 10.1128/JCM.40.9.3326-3333.2002 12202574 PMC130708

[4] Arias CA, Panesso D, Singh KV, Rice LB, Murray BE. Cotransfer of antibiotic resistance genes and a hylEfm-containing virulence plasmid in *Enterococcus faecium.* Antimicrob Agents Chemother. 2009;53:4240–6. 10.1128/AAC.00242-09 19667280 PMC2764207

[5] Benson MA, Ohneck EA, Ryan C, Alonzo F III, Smith H, Narechania A, Evolution of hypervirulence by a MRSA clone through acquisition of a transposable element. Mol Microbiol. 2014;93:664–81. 10.1111/mmi.12682 24962815 PMC4127135

[6] Stamatakis A. RAxML-VI-HPC: maximum likelihood-based phylogenetic analyses with thousands of taxa and mixed models. Bioinformatics. 2006;22:2688–90. 10.1093/bioinformatics/btl446 16928733

[7] Bouhss A, Josseaume N, Severin A, Tabei K, Hugonnet JE, Shlaes D, Synthesis of the L-alanyl-L-alanine cross-bridge of *Enterococcus faecalis* peptidoglycan. J Biol Chem. 2002;277:45935–41. 10.1074/jbc.M207449200 12324463

[8] Kos VN, Desjardins CA, Griggs A, Cerqueira G, Van Tonder A, Holden MT, Comparative genomics of vancomycin-resistant *Staphylococcus aureus* strains and their positions within the clade most commonly associated with methicillin-resistant *S. aureus* hospital-acquired infection in the United States. MBio. 2012;3:e00112–12. 10.1128/mBio.00112-12 22617140 PMC3372964

[9] Arbeloa A, Hugonnet JE, Sentilhes AC, Josseaume N, Dubost L, Monsempes C, Synthesis of mosaic peptidoglycan cross-bridges by hybrid peptidoglycan assembly pathways in gram-positive bacteria. J Biol Chem. 2004;279:41546–56. 10.1074/jbc.M407149200 15280360

[10] Nübel U, Roumagnac P, Feldkamp M, Song JH, Ko KS, Huang YC, Frequent emergence and limited geographic dispersal of methicillin-resistant *Staphylococcus aureus.* Proc Natl Acad Sci U S A. 2008;105:14130–5. 10.1073/pnas.0804178105 18772392 PMC2544590

[11] Caboclo RM, Cavalcante FS, Iorio NL, Schuenck RP, Olendzki AN, Felix MJ, Methicillin-resistant *Staphylococcus aureus* in Rio de Janeiro hospitals: dissemination of the USA400/ST1 and USA800/ST5 SCCmec type IV and USA100/ST5 SCCmec type II lineages in a public institution and polyclonal presence in a private one. Am J Infect Control. 2013;41:e21–6. 10.1016/j.ajic.2012.08.008 23261682

[12] Dabul AN, Kos VN, Gilmore MS, Camargo IL. Draft genome sequence of methicillin-resistant *Staphylococcus aureus* strain SA16, representative of an endemic clone from a Brazilian hospital. Genome Announc. 2013;1:e00754–13. 10.1128/genomeA.00754-13 PMC3778207 24051324

[13] Teixeira MM, Araujo MC, Silva-Carvalho MC, Beltrame CO, Oliveira CC, Figueiredo AM, Emergence of clonal complex 5 (CC5) methicillin-resistant *Staphylococcus aureus* (MRSA) isolates susceptible to trimethoprim-sulfamethoxazole in a Brazilian hospital. Braz J Med Biol Res. 2012;45:637–43. 10.1590/S0100-879X2012007500065 22527128 PMC3854277

[14] Hiramatsu K, Aritaka N, Hanaki H, Kawasaki S, Hosoda Y, Hori S, Dissemination in Japanese hospitals of strains of *Staphylococcus aureus* heterogeneously resistant to vancomycin. Lancet. 1997;350:1670–3 . 10.1016/S0140-6736(97)07324-8 9400512

[15] Arthur M, Depardieu F, Cabanie L, Reynolds P, Courvalin P. Requirement of the VanY and VanX D,D-peptidases for glycopeptide resistance in enterococci. Mol Microbiol. 1998;30:819–30 . 10.1046/j.1365-2958.1998.01114.x 10094630

