# Supplementary material for: Methicillin-Susceptible, Vancomycin-Resistant Staphylococcus aureus, Brazil
Source: Emerg Infect Dis. 2015 Oct;21(10):1844–8. doi: 10.3201/eid2110.141914 (PMC4593430; doi:10.3201/eid2110.141914)
Supplement: Technical Appendix — Analysis of methicillin-susceptible, vancomycin-resistant Staphylococcus aureus, Brazil. [file 14-1914-Techapp-s1.pdf]

Article DOI: http://dx.doi.org/10.3201/eid2110.141914

Methicillin-Susceptible, Vancomycin-Resistant *Staphylococcus aureus*, Brazil

Technical Appendix

Case-Patient Summary

The patient was a 35-year-old man with mycosis fungoides, cocaine addiction, diabetes mellitus, and a history of repetitive skin and soft tissue infections. He was first hospitalized and treated for leg cellulitis in November 2011 and readmitted for recurrent skin and soft tissue infections and worsening concurrent conditions in June 2012. During his hospitalization, repetitive febrile episodes developed, and he had blood cultures positive for different *Staphylococcus aureus* isolates. The clinical course of the patient, *Staphylococcus aureus* isolates, and antimicrobial drugs provided are summarized in Technical Appendix Figure 1. Further details can be found in a prior publication by Rossi et al. (*1*).

Genome Sequencing

MiSeq assembly was performed by using ABySS (*2*), and PacBio assembly was performed by using the HGAP2 v2.1 de novo assembly pipeline (Pacific Biosciences, Menlo Park, CA, USA). Comparison of single-nucleotide polymorphisms (SNPs) between genomes used in this study was performed by using the short read alignment to the *S. aureus* genome for strain N315 as a reference and the Burrows-Wheeler Alignment tool (http://bio-bwa.sourceforge.net). SNP calls were detected by using samtools (http://samtools.sourceforge.net), and SNPs were identified as high quality if they were unambiguous and had a q score $\geq$20. For preassembled genomes available from public databases, we used whole-genome alignment with reference to the N315 genome by using the show-snps utility of NUCmer (http://mummer.sourceforge.net). We created phylogenetic datasets by combining results of both SNP calling techniques above. We excluded potentially repeated regions from the reference genome that had >80% nucleotide similarity over 100 bp on the basis of BLAST

(http://blast.ncbi.nlm.nih.gov/Blast.cgi) of the genome against itself. All locations in the genome annotated as mobile genetic elements were also excluded.

Phylogenetic Methods

Maximum-likelihood phylogenies were constructed by using the POSIX-threads version of RAxML v8.0.19 (*3*). For SNP data, we used an ascertainment bias correction and a general time-reversible substitution model accounting for among-site rate heterogeneity by using the gamma distribution and 4 rate categories (ASC_GTRGAMMA model) for 100 individual searches with maximum parsimony random-addition starting trees. Node support was evaluated with 1,000 nonparametric bootstrap pseudoreplicates and filtering the optimal maximum-likelihood tree through the bootstrap trees so that node support values shown indicate the percentage proportion of bootstrap trees that contained a given internode branch.

Peptidoglycan Precursor and Cell Wall Analyses

Extraction of peptidoglycan precursors was performed as described (*4*). Separation of precursors by reversed-phase, high-performance liquid chromatography was conducted by using a C18 column (Nucleosil 4.6 × 250 mm; Macherey-Nagel, Hoerdt, France). Peaks were collected and precursors were identified by mass spectrometry (Qstar Pulsar I; Applied Biosystems, Courtaboeuf, France) (*4*). The peptide moiety of the precursors was sequenced by tandem mass spectrometry (*4*). Relative abundance of precursors was estimated by the percentage of the integrate peak area at 262 nm. Peptidoglycan was prepared as described (*5*), and covalently attached proteins were removed from peptidoglycan by digestion with pronase and trypsin. Muropeptides were obtained by digestion with lysozyme and mutanolysin. The ether bond internal to N-acetylmuramic acid was cleaved with 3% ammonia, and the resulting lactoyl peptides were separated by reversed-phase, high-performance liquid chromatography for sequencing by tandem mass spectrometry (Qstar Pulsar I).

References

1. Rossi F, Diaz L, Wollam A, Panesso D, Zhou Y, Rincon S, et al. Transferable vancomycin resistance in a community-associated MRSA lineage. N Engl J Med. 2014;370:1524–31. PubMed http://dx.doi.org/10.1056/NEJMoa1303359

2. Simpson JT, Wong K, Jackman SD, Schein JE, Jones SJ, Birol I. ABySS: a parallel assembler for short read sequence data. Genome Res. 2009;19:1117–23. PubMed http://dx.doi.org/10.1101/gr.089532.108

3. Stamatakis A. RAxML-VI-HPC: maximum likelihood-based phylogenetic analyses with thousands of taxa and mixed models. Bioinformatics. 2006;22:2688–90. PubMed http://dx.doi.org/10.1093/bioinformatics/btl446

4. Bouhss A, Josseaume N, Severin A, Tabei K, Hugonnet JE, Shlaes D, et al. Synthesis of the L-alanyl-L-alanine cross-bridge of *Enterococcus faecalis* peptidoglycan. J Biol Chem. 2002;277:45935–41. PubMed http://dx.doi.org/10.1074/jbc.M207449200

5. Arbeloa A, Hugonnet JE, Sentilhes AC, Josseaume N, Dubost L, Monsempes C, et al. Synthesis of mosaic peptidoglycan cross-bridges by hybrid peptidoglycan assembly pathways in gram-positive bacteria. J Biol Chem. 2004;279:41546–56. PubMed http://dx.doi.org/10.1074/jbc.M407149200

Technical Appendix Table 1. Genome statistics for *Staphylococcus aureus*, Brazil*

Strain	Coverage	No. contigs	Mean subread length, bp	Read length N50/assembly N50	NCBI Bioproject no.
VR-MSSA (HP022)	800×	1,437	NA	NA/189054 bp	PRJNA262896
VS-MSSA (HP023)	575×	1,438	NA	NA/91,499 bp	PRJNA262928
M1 (HP012)	250×	1,813	NA	NA/8,727 bp	PRJNA262670
M91 (HP013)	85×	1,808	NA	NA/46,912 bp	PRJNA262672
VR-MSSA (PacBio)	81.1×	9†	4,955	6,305 bp/ 2.04 Mbp	PRJNA262896

*NCBI, National Center for Biotechnology Information; VR-MSSA vancomycin-resistant, methicillin-susceptible *S. aureus*; NA, not applicable; VS-MSSA, vancomycin-susceptible, methicillin-susceptible *S.aureus*.
†Manual polishing and additional assembly resulted in 4 contigs (1 closed circular chromosome and 3 extrachromosomal elements).

Technical Appendix Table 2. Mass of muropeptide from vancomycin-susceptible and vancomycin-resistant, methicillin-susceptible *Staphylococcus aureus*, Brazil*

Strain (growth condition)	R substituent of muropeptide		Value	Monoisotopic mass of muropeptide, atomic mass units					
	R1	R2		Monomer, n = 0	Dimer, n = 1	Trimer, n = 1	Tetramer, n = 2	Pentamer, n = 3	Hexamer, n = 5
VS-MSSA	D-Ala- D-Ala	Gly_5	Calculated	844.40	1,599.76	2,355.12	3,110.47	3,865.83	4,621.18
			Observed	844.42	1,599.79	2,355.18	3,110.55	3,865.92	4,621.38
VR-MSSA (induced)†	D-Ala	H	Calculated	488.26	1,243.62	1,998.97	2,754.33	3,509.68	4,265.04
			Observed	488.25	1,243.6	1,998.97	2,754.34	3,509.71	4,265.08
	D-Ala	Gly_5	Calculated	773.37	1,528.72	2,284.08	3,039.44	3,794.79	4,550.14
			Observed	773.38	1,528.73	2,284.08	3,039.46	3,794.83	4,550.17

*VS-MSSA, vancomycin-susceptible, methicillin-susceptible *S.aureus*; VR-MSSA vancomycin-resistant, methicillin-susceptible *S.aureus*.
†Induction was performed with 10 µg/mL of vancomycin.

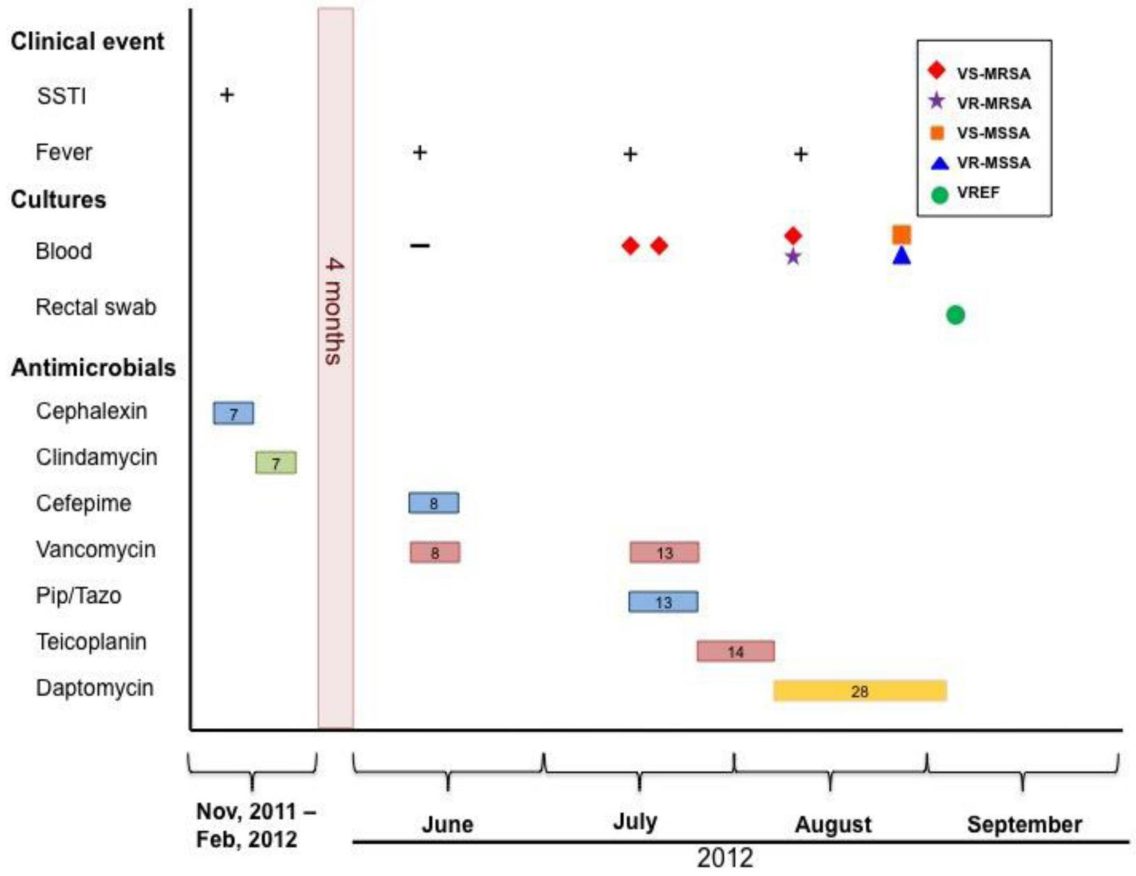

Technical Appendix Figure 1. Clinical course timeline of the patient, Brazil. Drugs used are indicated by colored rectangles: β-lactams in blue (cephalexin, cefepime, and piperacillin/tazobactam [Pip/Tazo]), clindamycin in green, glycopeptides in pink (vancomycin and teicoplanin), and daptomycin in yellow. The number in each rectangle corresponds to the number of days of treatment with the drug. Drugs are shown in the order in which they were added to therapy. The final days of hospitalization are not included. SSTI, skin and soft-tissue infection; VS-MRSA, vancomycin-susceptible, methicillin-resistant *Staphylococcus aureus*; VR-MRSA, vancomycin-resistant, methicillin-resistant *S. aureus*; VS-MSSA, vancomycin-susceptible, methicillin-susceptible *S.aureus*; VR-MSSA vancomycin-resistant, methicillin-susceptible *S.aureus*; VREF, vancomycin-resistant *Enterococcus faecalis*.

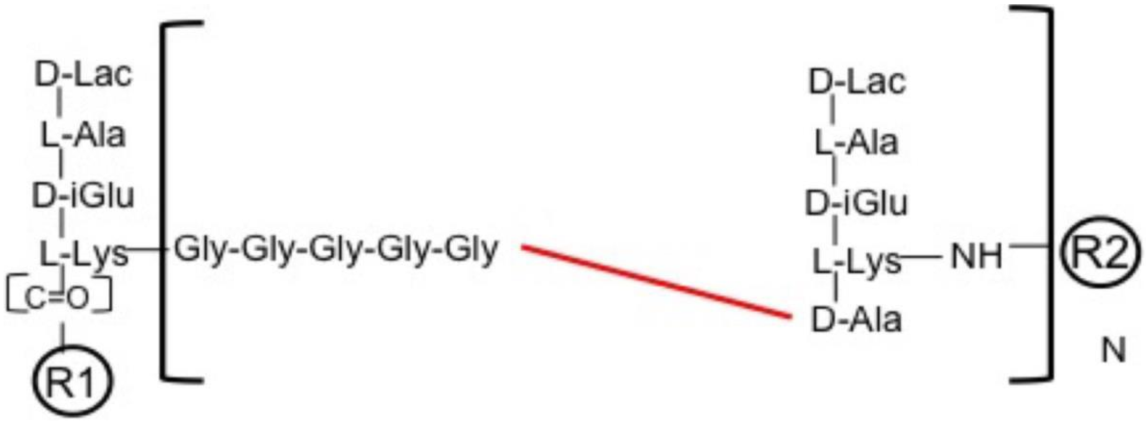

Technical Appendix Figure 2. Diversity in the structure of muropeptides from *Staphylococcus aureus*, Brazil. Diversity of muropeptides is generated by variations at the C-terminus (R_1 = OH or D-Ala^{4}- D-Ala^{5}), at the N terminus (R_2 = H or D-Gly$_5$) and by the extent of oligomerization (from N = 0 for monomers to N = 6 for heptamers).

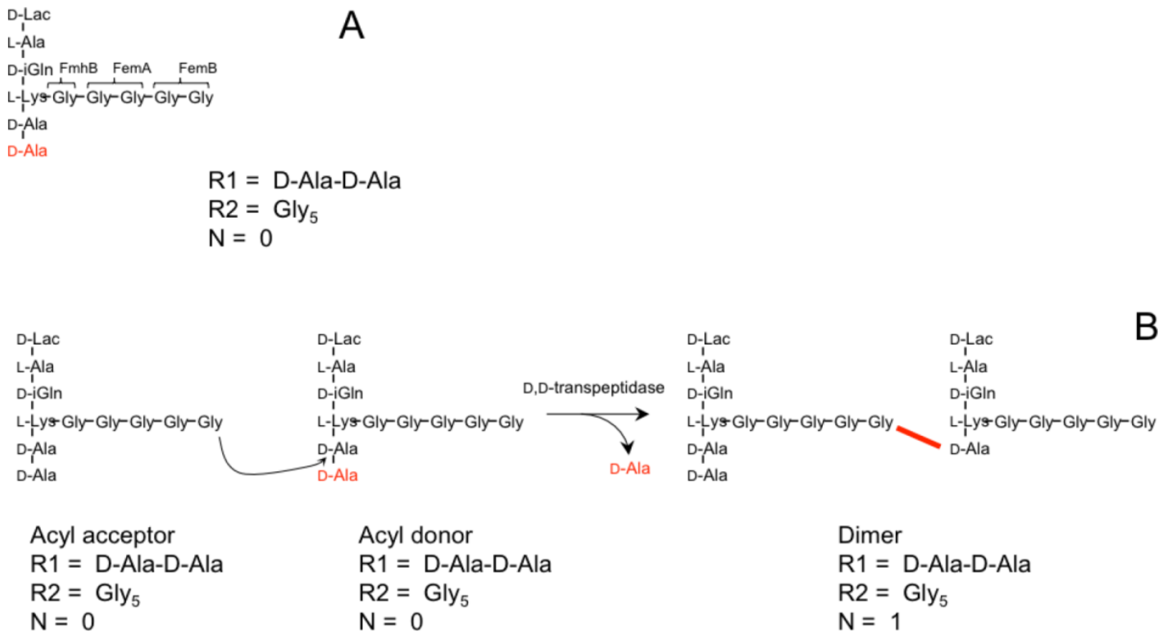

Technical Appendix Figure 3. Muropeptides from vancomycin-susceptible, methicillin-susceptible *Staphylococcus aureus*, Brazil. A) Main monomers. The side-chain is assembled by aminoacyl transferases of the Fem family that sequentially add the first (FmhB), second, and third (FemA), and fourth and fifth (FemB) Gly residues. B) Dimer generated by D,D-transpeptidation. The D,D-transpeptidases cleave the D-Ala^{4}- D-Ala^{5} peptide bond of the acyl donor and link the carbonyl of D-Ala^{4} to amino group located at the extremity of the side chain of the acyl acceptor.

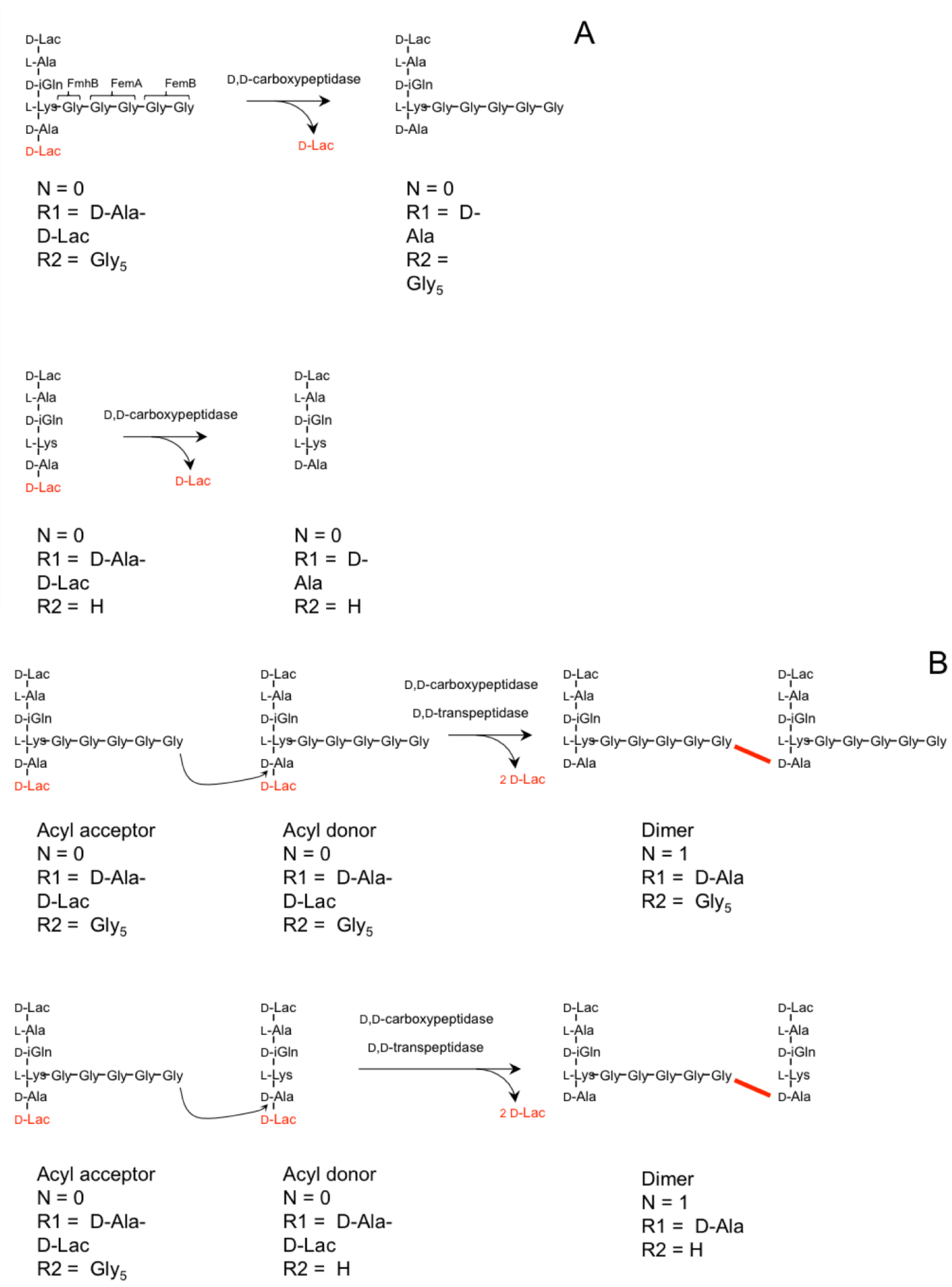

Technical Appendix Figure 4. Muropeptides from vancomycin-resistant, methicillin-susceptible *Staphylococcus aureus* grown in the presence of 10 µg/mL vancomycin, Brazil. A) Main monomers. The C-terminal D-Lac is cleaved by D,D-carboxypeptidase and is not found in mature

peptidoglycan. Most (62%) of the muropeptide monomers did not contain any side-chain (R_2 = H instead of Gly_5) because of impaired activity of FmhB with D-Lac ending precursors. B) Dimer generated by D,D-transpeptidation. All cross-links contain Gly_5 because unsubstituted stem peptides (R_2 = H) are not used as acyl acceptors by D,D-transpeptidases.

Tree

```
#NEXUS

begin taxa;

dimensions ntax = 50;

taxlabels

VRS3a

HP022

HP023

SaED98

SaMSHR1132

Sa08BA02176

SaST398

SaTCH60

SaMRSA252

SaJKD6159

SaLGA251\

SaED133

SaRF122

SaMW2

SaMSSA476

Sa1181997
```

SaT0131

SaJKD6008

SaTW20

VSSA

VRSA

SaUSA300TCH1516

SaUSA300FPR3757

SaNewman

USA500

SaCOL

SaNCTC8325

SaVC40

SaN315

SaECTR2

Sa16

Sa0402981

VRS10

VRS4

VRS5

VRS7

SaJH1

SaJH9

VRS11b

VRS11a

VRS6

VRS8

VRS9

SaMu50

SaMu3

VRS1

VRS2

SaST228

HP013

HP012

;

end;

begin trees;

tree tree_1 = [&R] (((VRS3a:8.244799231807832E-4,((((HP022:3.967364433824444E-6,HP023:2.250523383219172E-5):3.411632724734676E-4,(SaED98:9.703221642786964E-4,((SaMSHR1132:0.5632346423186245,(((Sa08BA02176:5.105885699270055E-4,SaST398:5.538524020539065E-4):0.023187580060007828,(SaTCH60:0.0018606904107206922,SaMRSA252:0.0023361910795642177):0.0228694576846738):0.0208677517984276,(SaJKD6159:0.043048096207328516,((SaLGA251:0.012751949141110169,SaED133:0.014657491012749579):0.0077023084595625556,SaRF122:0.020706149045281765):0.005715274275517768):0.005352806350899746):0.014270643684360873):0.004215184179548835,(((SaMW2:8.148223984244278E-4,SaMSSA476:9.399261875994422E-4):0.011723988543628189,Sa1181997:0.012031146247175885):0.003472808070423307,(((SaT0131:0.0011290280300672703,SaJKD6008:4.616415737154414E-4):2.4208588870377014E-4,SaTW20:5.876500448633564E-

4):0.011876794547025669,(((VSSA:2.3017304230714717E-
4,VRSA:4.574144121631048E-
4):0.0017108374225468953,(SaUSA300TCH1516:5.690969067851791E-
5,SaUSA300FPR3757:7.414423176218751E-
5):0.0012672330396069006):5.448881866674404E-
4,(((SaNewman:9.578627969952902E-
4,USA500:0.0015003244427622417):4.515479337227684E-
4,SaCOL:0.001221232134307728):3.865704697744097E-
5,(SaNCTC8325:3.3883278053041993E-4,SaVC40:3.1202602748559656E-
4):8.030801728358954E-4):1.5673146668809766E-
4):0.002729247117285292):0.009758518105999302):0.004378187341032908):0.011153
156773090636):1.0540166871535802E-4):2.024831647401745E-
5,SaN315:1.8085112342770083E-4):3.89970547585776E-
6,(SaECTR2:1.7884162073543815E-4,((Sa16:3.063593398554345E-
4,Sa0402981:1.4522808953175689E-4):5.25613854399233E-
6,(VRS10:1.463713478899369E-4,(((((VRS4:1.2530559741665855E-
4,((VRS5:2.535180723808072E-5,VRS7:1.038618732320479E-
5):1.2164112975865705E-4,(SaJH1:1.83460916665427E-
6,SaJH9:3.441460745719972E-5):9.49510315513687E-5):2.218797396207839E-
5):6.151791798841402E-5,(VRS11b:1.720642232484988E-
5,VRS11a:1.323680283108371E-5):1.7233451943842544E-4):4.229759968813017E-
4,(VRS6:3.8725918864612193E-4,(VRS8:6.861586445225702E-
4,(VRS9:6.449336162285586E-4,(SaMu50:2.516166638565688E-
5,SaMu3:1.984252037642552E-5):0.0010528299728617904):1.6565223374051232E-
4):6.191284786351566E-5):2.843936663916666E-4):2.1983545993385344E-
4,VRS1:4.787935705512973E-4):1.0877097026918476E-
4,VRS2:3.636107322352297E-4):1.195311846552679E-5):2.663733436769372E-
5):3.077751783223781E-5):1.261544142693425E-4):1.666869357284407E-
4):5.126069298913468E-4,SaST228:0.0012028662762011897):4.5280891172319815E-
4,HP013:8.712211399490469E-5,HP012:4.732197689535684E-4);

end;

Matrix

Large file, available from the authors.
